# Early Lung Function and Future Asthma

**DOI:** 10.3389/fped.2019.00253

**Published:** 2019-06-19

**Authors:** Manuel Sánchez-Solís

**Affiliations:** ^1^Department of Pediatric, Hospital Universitario Virgen de la Arrixaca, Murcia, Spain; ^2^Biomedical Research Institute of Murcia (IMIB), Palmar, Spain; ^3^Department of Surgery, Pediatric, Obstetric and Gynaecology, University of Murcia, Murcia, Spain

**Keywords:** wheeze, asthma, lung function, bronchial (airway) hyperresponsiveness, asthma risk factors

## Abstract

Asthmatic adults with lower lung function have been described as having had this worse condition early in life. Lung function is reduced in children with persistent asthma and continues low throughout adult life. The challenge is to know if impaired lung function is a risk factor of asthma, as a consequence of special congenital characteristics of the airways, or whether asthmatic patients suffer a loss in lung function as early as 9 years of age as a consequence of very precocious remodeling of the airways. The loss is so early in life that it is probably a congenital characteristic, however there is not a cut-off point with clinical interest to predict risk of asthma later in life. There are contradictory results regarding whether asthmatic children lose lung function as a consequence of the airway remodeling by the illness itself. This aspect seemed to be shown for children at risk—the offspring of asthmatic mothers. The early BHR seems to be very frequent even in healthy infants, but is probably not a risk factor for asthma years later; except in the offspring of asthmatic mothers in which it has been shown. There are still many uncertainties in this field; so, more research is needed in order to better understand the pathophysiology of asthma, the early risk factors and to design new therapeutic targets and early interventions to change the natural history of the disease.

The Global Initiative for Asthma (GINA) (1) defines asthma as “a heterogeneous disease, generally characterized by chronic inflammation of the respiratory tract. It is defined by the history of symptoms such as wheezing, dyspnoea, chest tightness, and cough that vary with time and intensity, together with a variable limitation of the expiratory air flow.” Therefore, from the point of view of lung function, the essential characteristic in asthma is the variable limitation of the expiratory flow. In children, and especially in infants, the assessment of this limitation is not easy and not only due to a lack of collaboration, but also due to the lack of consensus regarding the best technique for clinical use, as well as the fact that there are no good reference equations available (2). Despite these limitations, just 3 years ago, the official practical guides by the ATS on recurrent and persistent wheezing recommended lung function studies in infants by means of the raised volume rapid thoracoabdominal compression (RVRTC) technique in order to assess its usefulness for better patient management (3).

It has been described that adult asthmatics who have a reduced lung function have had this condition since at least the age of 9 years (4). A recent metanalysis showed that serious infant asthma may be accompanied by a reduction in lung function that persists into adult life (5). The challenge is to know if the altered lung function is a risk factor for asthma, as a consequence of congenital characteristics in the airways, or if asthmatic patients suffer from a loss in lung function as early as 9 years of age, or even before, as a consequence of an early remodeling of the airways.

Cohort studies offer the major advantage of enabling the factors that precede the development of asthma to be studied and, therefore, help us to establish risk criteria. With regard to the development of lung function and asthma throughout life what has been learnt from cohort studies can be approached from different points of view:

(1) Is congenital reduced lung function a risk factor for the future development of asthma?

(2) Does asthma itself cause a loss in lung function in an early stage of life?

(3) Is the development of bronchial hyperresponsiveness (BHR) a risk factor for future asthma?

## Is Congenital Reduced Lung Function a Risk Factor for Future Asthma?

Several cohort studies have demonstrated that altered lung function in the first weeks of life and before the first wheezing episode is subsequently associated with asthma (Table 1) (6–12, 14, 16–18).

**Table 1:** Relationship of lung function measured in new-borns and infants and its relationship with asthma.

**Author**	**Cohort**	**Age of first lung function test**	***N***	**Main results**
Martinez et al. (6)	Tucson children's respiratory study	2.4 ± 2 months	124	The children whose VmaxFRC was in the lowest tertile had a greater risk of recurrent wheezing during the first 3 years of life in comparison to those in the upper two tertiles (OR = 4.6; 95%CI 1.3 to 16.1, *p* = 0.012).
Martinez et al. (7)				The children with transient early wheezing have lower V_maxFRC_, measured before 6 months of age than the healthy children (mean = 70.6 mL/s; 95%CI 52.2 mL/s to 93.8 mL/s vs. mean = 123.3 mL/s; 95%CI 110.0 mL/S to 138.0 mL/s; *p* < 0.001) and this alteration continues to 6 years of age. Children with persistent wheezing do not have worse V_maxFRC_, measured before 6 months of age, than healthy children, but do so if it is reduced at 6 years of age (mean = 1,262.1 mL/s; 95%CI(1,217.4 mL/s to 1308.1 mL/s vs. mean = 1,069.7 mL/s; 95%CI 906.9 mL/s to 1,146.5 mL/s; *p* < 0.001).
Stern et al (8)				The children who, before 6 months of age, had a V_maxFRC_ value in the lowest tertile, showed, at 11, 16, and 22 years of age, a significantly worse lung function (in terms of FEV_1_, FEV_1_/FVC, and FEF_25−75_). The V_maxFRC_ measured before 6 months correlates significantly with FEV_1_/FVC and FEF_25−75_, at the ages of 11, 16, and 22 years.
Young et al. (9)	Perth cohort	1 month	243	The patients who, at 1 month of life, had a limited flow (understood as when the forced expiratory flow did not exceed tidal flow at functional residual capacity) had a greater risk of being diagnosed with asthma at 2 years of age (OR = 7.4; 95%CI 1.4 to 35.2).
Turner et al. (10)				A lower V_maxFRC_ at 1 month of life is an independent factor for persistent wheezing at 11 years of age (Hazard Ratio = 0.18; IC95% 0.00–0.73; *p* = 0.03). On the other hand, the mean of the z-scores of V_maxFRC_ and FEF_25−75_ at 6 and 11 years, was consistently lower in the group with persistent wheezing compared to the group that never presented wheezing (−0.57 ± 0.91 vs. 0.19 ± 0.88; *p* = 0.001).
Turner et al. (11)				At 18 years, the children with flow limitation before 1 month of life, had worse lung function (mean reduction 17.4%; 95%CI 7.8% to 27.1%; *p* < 0.001), and have greater risk of presenting wheezing in the previous year (OR 3.3 (95% CI 1.0 to 11.1), but it was not found that reduced lung function is related with asthma at that age (% change in V_maxFRC_/FEF_25−75_ and asthma ever = -6.0%; 95%IC −12.2 to +0.3; *p* = 0.061).
Owens et al. (12)				At 24 years of age, the asthmatic patients (current asthma) had a neonatal V_maxFRC_ significantly lower compared to those who had never been asthmatics (never asthma) (% of predicted = 68.7%;95% C: 47.7 to 89.7% vs. 109.9%;95% CI 100.6 to 119%; *p* = 0.001). The children with V_maxFRC_ in the lowest quartile (66.3% of predicted), had a greater risk of asthma at 24 years of age (OR = 5.1; 95%CI 2 to 13.2; *p* = 0.001).
Pike et al. (13)	Southampton women's survey (SWS)	5–14 weeks	V_maxFRC_: 138 FEV_0.4_: 108	At 3 years of age the children with wheeze had, in the first weeks of life, a significantly lower V_maxFRC_ than those who never presented wheezing (difference −23.2%; 95%CI −36.4% to −7.3%; *p* = 0.006). At 3 years of age the non-atopic children with wheezing had, in the first weeks of life, a significantly lower FEV_0.4_ than those who never had wheezing (difference −11.0%; 95%CI −19.2% to −2.0%; *p* = 0.02).
Collins et al. (14)				Using the classification of phenotypes proposed by the Tucson Children's Respiratory Study (TCRS), the patients with transient wheezing had worse V_maxFRC_ measured at 5–14 weeks (ratio geometric mean: 0.71; 95%CI 0.58 to 0.88; but not those with persistent wheezing ratio geometric mean: 0.67; 0.43–1.05). However, when they used the phenotype classification criteria of the Avon Longitudinal Study of Parents and Children (ALSPAC), both the transient (ratio geometric mean: 0.64; 95%CI 0.50 to 0.82) as well as the persistent wheezers (ratio geometric mean: 0.54; 95%CI 0.32 to 0.91) had worse V_maxFRC_ measured at 5–14 weeks. Moreover, considering the ALSPAC criteria both the persistent wheezers as well as the transient wheezers, had worse lung function (FEV_1_, FEV_1_/FVC, and FEF_25−−75_; *p* < 0.05) at 6 years. On the other hand, with the TCRS criteria, only the persistent wheezers showed a permanent deficit in lung function (FEV_1_ and FEF_25−75_; *p* < 0.05) at 6 years.
Brooke et al. (15)				At 6 years of age, the authors found that a low FEV_0.4_ is a risk factor for asthma (ratio geometric mean. 0.83; 95%CI 0.71 to 0.97; *p* < 0.05). At this age, a low V_maxFRC_ is related only with the transient phenotype (ratio geometric mean: 0.71; 95%CI 0.58 to 0.88).
Bisgaard et al. (16)	Copenhagen prospective studies on asthma in childhood (COPSAC_2000_)	1 month	403	A lower FEF_50_ value at 1 month of life represents a greater risk of asthma at 7 years (OR 1.57; 95%CI 1.04 to 2.37; *p* = 0.03). The asthmatics had a significantly lower neonatal FEF_50_ (z score difference: −0.34; *p* < 0.03). A similar trend was observed for FEV_0.5_ (OR 1.41; 95%CI 0.94 to 2.10; *p* < 0.09. *z*-score difference −0.29; *p* < 0.07). Asthma at 7 years is an independent factor for the loss in FEF_50_ (*z*-score from 1 m to 7 y = −0.69; 95%CI −1.01 to −0.37; *p* = 0.0001) and in FEV_0.5_/ FEV1 (*z*-score from 1 m to 7 y = −0.62; 95%CI −0.94 to −0.29; *p* < 0.0002).
Halllas et al. (17)				Asthmatic children had a reduction in lung function from birth until 13 years compared with non-asthmatics; without progression or regression during that period of time (*z*-score difference of FEV_z_ −0.31; 95%CI −0.47 to −0.15; *p* < 0.01 and *z*-score difference of MMEFz = −0.44; 95% CI −0.60 to −0.27; *p* < 0.001).No interaction was found between asthma and the age at which lung function is measured (FEVz *z*-score: estimation of the interaction = −0.00; 95%CI −0.02 to +0.02; *p* = 0.82 and MMEFz z-score: estimation of the interaction: −0.01; 95%CI −0.03 to +0.01; *p* = 0.34). No variation was found in lung function in the first 13 years of life in the asthmatic patients (difference/year in the *z*-score of FEVz: +0.02; 95%CI −0.02 to +0.07; *p* = 0.36); as well as that there does not appear to be, due to the fact that the asthma remits (z-score difference of FEVz: +0.02; 95%CI −0.03 to +0.06; *p* = 0.44).

*Evolution of lung function of asthmatics during infancy and adolescence. Cohort studies*.

The Tucson Children's Respiratory Study (TCRS) was the first study designed to clarify if respiratory infections in the lower airways led to lesser lung function, or whether an early deteriorated lung function—prior to the first airway infection—increased the risk of wheezing. In that prospective study, the pre-morbid total respiratory conductance was measured in 124 neonates and the authors informed that the risk of having a wheezing episode was 3.7 times greater (95%CI 0.9 to 15.5; *p* = 0.06) among infants whose measurements were in the lowest tertile, in comparison with those infants with values in the highest two tertiles (18). This led the authors to suggest that the initial diameter of the airway and other specific characteristics of the lung parenchyma could predispose to wheezing during infancy with airway respiratory viruses. Moreover, the same study showed, by evaluating lung function through Rapid Thoracoabdominal Compression (RTC) to obtain the maximum flow to the functional residual capacity (V_maxFRC_), that those children whose V_maxFRC_ was in the lowest tertile had a greater risk of recurrent wheezing during the first 3 years of life in comparison with those in the upper two tertiles (OR = 4.6; 95%CI 1.3 to 16.1, *p* = 0.012) (6).

Several years later, in 1995 and when the cohort had reached 6 years of age, the study published three phenotypes of children with wheeze: (1) transient wheezing; (2) late-onset wheezing; and (3) persistent wheezing. It showed a low pre-morbid V_maxFRC_ measured before the age of 6 months, only in the phenotype of transient wheezing. However, at 6 years of age it was reduced both in those children with the transient wheezing phenotype, as well as in those that presented the persistent phenotype (7) (Table 1). This suggests that some children have a congenital deteriorated lung function (phenotype of transient wheezing), but that, in other children, the lung function is deteriorated very early on; before the age of 6 years of life.

In 2007, the result of the follow-up of lung function to the age of 22 years of the Tucson cohort was published (8): the authors found that infant lung function correlated with the measurements of the expiratory volume and flows (FEV_1_, FEF_25−75_, and FEV_1_/FVC) between 11 and 22 years and that the patients in the lowest quartile of infant lung function had a ratio of FEV_1_, FEF_25−75_, and FEV_1_/FVC lower at 22 years of age than those in the upper three quartiles together. In the words of the authors: this finding suggests that those persons with obstruction in the airflow at birth will have greater probabilities of remaining in the lowest end of the distribution until adult life (8). However, they did not find that patients with the lowest V_maxFRC_ had a greater risk of asthma at 11, 16, or 22 years. The authors even speculated about the possibility that these infants with low lung function at birth with transient wheezing may be in danger of developing chronic obstructive pulmonary disease and that the prevention of this disease could begin intrauterine.

In summary, we could state that the principal findings of this cohort are that: (1) a group of infants has a congenitally diminished lung function; (2) said alteration in lung function persists until adult life; and (3) said functional alteration is a risk factor for wheezing in the first 3 years of life, but is not a risk factor for asthma, at least at 11, 16, and 22 years of age.

The Perth cohort (9–12) also showed a relationship between low pre-morbid lung function and the subsequent diagnosis of asthma (Table 1): they found that infants with flow limitation (which they defined as when the value of V_maxFRC_ is not greater than the current volume at functional residual capacity) have a greater risk of asthma at 2 years of age (9). On the other hand, at 11 years of age, the V_maxFRC_ at 1 month of life is an independent risk factor for persistent asthma (Hazard Ratio: 0.18; 95%IC 0.00 to 0.73; *p* = 0.03) and the lung function z-score (i.e., V_maxFRC_ at 1 month and FEF_25−75_ at 6 and 11 years of age) is significantly lower in the group with persistent asthma compared to the non-asthmatic group (*p* = 0.001) (10). These results contradict those described in the Tucson study, since the Australian study suggests that the development of persistent asthma until school age is, at least partially, predetermined by a congenitally affected lung function.

That same study found that, at 18 years of age (11), those children with flow limitation before 1 month of life, had worse lung function and, additionally, greater risk of presenting wheezing in the previous year (Table 1). However, they did not find reduced lung function to be related with persistent asthma; although the trend seems clear and was found to be very near to statistical significance (% change in V_maxFRC_/FEF_25−75_–asthma ever = −6.0%; 95%IC −12.2 to +0.3; *p* = 0.061). In fact, some years later in this same cohort it was found, at the age of 24 years, that the asthmatic patients had a neonatal V_maxFRC_ significantly lower compared to those who had never been asthmatic (% of predicted = 68.7%; 95% C: 47.7 to 89.7% vs. 109.9%; 95% CI 100.6 to 119%; *p* = 0.001) and also that those neonates with V_maxFRC_ in the lowest quartile (66.3% of predicted), had a greater risk of asthma at 24 years (OR = 5.1; 95%CI 2 to 13.2; *p* = 0.001) (12). Another interesting aspect of this evaluation at 24 years of age is that the patients whose asthma remitted had a neonatal lung function significantly better that those who suffered from persistent asthma (mean V_maxFRC_ = 105%; 95% CI: 88–123% vs. 69%; 95% CI: 53–84%; *P* = 0.003) (12).

Thus, the Perth cohort shows that neonatal reduction in lung function is a risk factor for persistent asthma throughout life and that this functional respiratory limitation is also maintained, at least, until youth. The authors themselves (12) suggest that “*subjects with more significant asthma symptoms had a defect in lung development or growth in utero or very early in life that persisted as a reduction in lung function into adulthood.”*

It is not easy to analyse the reason for such contradictory results between the Tucson and Perth cohorts. The limited number of patients recruited is probably responsible. It should be noted that the Tucson cohort, which did not find such differences, in the measurements taken around 2 months of life the group of those that will have persistent asthma is only represented by 16 patients compared to the 67 children without wheezing. In the Perth cohort, the number of patients in the persistent wheezing group was 17 vs. the 67 who had never had wheezing. On the other hand, the phenotype classification criteria may not coincide exactly between the two studies. In this sense, Collins et al. (14) performed, with the data from the Southampton Women's Survey (SWS) cohort, the comparative analysis among the six phenotypes proposed in the Avon Longitudinal Study of Parents and Children (ALSPAC) (19) with the four phenotypes proposed by the TCRS and showed that using the ALSPAC criteria both the children with transient wheezing as well as those who suffered from persistent wheezing, had a low V_maxFRC_ in the first weeks of life, but that using the TCRS criteria, only the transient ones showed that deficit. The persistent group in the TCRS study included two ALSPAC sub-groups: those with very early-onset wheezing, around 6 months of age, and those who began shortly after, around 18 months. These latter ones, named as the intermediate-onset group by ALSPAC, did not have lung function alterations in the first weeks of life [according to the study by Collins et al. (19)], whilst the persistent wheezing group did. An overrepresentation of “intermediate-onset” patients in the “persistent” group of the TCRS study could explain the absence of differences in the V_maxFRC_, in the first weeks of life, when the persistent wheezing group is compared with the non-wheezers. Another argument in favor of this possibility is that, from 6 years of age, the TCRS persistent wheezers (7) and both the intermediate-onset and the persistent wheezers in the ALSPAC (19), had a limited lung function compared to the group that never had wheeze. Thus, are these incongruences merely a problem of classification criteria and of sample size? The answer is probably yes.

The Southampton Women's Survey cohort (13) was the first to measure the forced expiratory volume in 0.4 s (FEV_0.4_) by means of the RVRTC technique, as well as the V_maxFRC_. The authors found, once again, a statistically significant association between low V_maxFRC_ with suffering wheezing until 3 years of age (Table 1). On the other hand, it was the non-atopic children who still had wheezing into their third year of life who had the lowest FEV_0.4_, measured between 5 and 14 weeks of life; the values of FEV_0.4_ in the atopic children were not significantly different among those who had had wheezing or had not (*p* = 0.4). This suggests that the caliber of the lower airway, in some children, may be a risk factor for asthma, independently of the atopic status. That cohort also showed that, at 6 years of age, a low neonatal FEV_0.4_ is a risk factor for asthma. Although, at that age, a low neonatal V_maxFRC_ is associated only with the transient phenotype (15).

The Copenhagen Prospective Studies on Asthma in Childhood (COPSAC) cohort (16) found that those patients diagnosed as asthmatic at 7 years of age had a lower lung function when they were neonates, specifically the forced expiratory flow at 50% of the forced vital capacity (FEF_50_); moreover, the drop in the neonatal FEF_50_ is a risk factor for asthma at that age (Table 1). Similar trends were observed with the FEV_0.5_. The Danish cohort did not classify the patients by phenotype. Another important finding was that at 7 years, the asthmatic patients showed a reduced lung function in terms of FVC (*p* = 0.008), FEV_1_ (*p* < 0.0001), FEV_1_/FVC (*p* = 0.0001), and FEF_50_ (*p* < 0.0001). The recent publication of the results obtained with the follow-up until 13 years (17) showed similar results: the children that developed asthma already had a reduced lung function as neonates and this remains altered to the age of 13 years compared to the non-asthmatics (Table 1). The COPSAC study (16, 17) was designed to research into the development of asthma in children at risk; it is a prospective clinical study of a cohort recruited at birth because their mothers had a history of asthma diagnosed by a physician after the age of 7 years; that may affect the external validity of the conclusions. Its principal strength, however, lies in the fact that the number of neonates whose lung function was measured was 403.

Thus, with the exception of the Tucson Children's Respiratory Study, the rest of the cohorts coincide in that the lung function was worse, measured in the first months of life, in those children who developed asthma, and that said lung function is determined very early on, probably congenitally, and remains altered throughout life; at least into their youth. It is important that it has not been described whether a cut-off point with clinical interest exists to predict the future asthma risk.

## What Factors Are Related With Early Loss in Lung Function?

Several factors related with the early loss in lung function have been described:

1. Prematurity. It has been described that lung function in infants born pre-term is diminished (20, 21) and that this reduction probably remains throughout their life, at least between the ages of 4 and 19 years (22).
2. Pre-natal exposure to Tobacco. The Perth cohort found that the V_maxFRC_ of those infants whose mothers smoked during the pregnancy was significantly lower than that of the children of non-smoker mothers (*p* = 0.05) (23). The COPSAC cohort studied the factors that determine neonatal lung function and found a 7% loss in the FEV_0.5_ in the children of smoker mothers (ß = 0.930; 95%CI 0.878 to 0.985; *p* = 0.013) (24).
3. Increase in weight in the first months of life. The COPSAC cohort found that the neonatal FEV_0.5_ was 14% lower in children with a body mass index in the uppermost quartile (24). On the other hand, the lung function at 6 weeks of life was not significantly different in babies born small for their gestational age compared to new-borns with appropriate weight for their gestational age (25). Perhaps the weight at a specific moment is not as important as its overly rapid increase, since the Perth cohort found that during the first year of life, the V_maxFRC_ was 10.39 mL lower per Kg of weight of the child (23) and they described that the gain in V_maxFRC_ in the first year of life was inversely related to the weight gain in that same period of time (26). One recent publication (27) has studied the relationship between fetal weight gain (estimated by means of ultrasound) and weight gain during the first year of life, with the lung function measured at 10 years of age. That study found a close relationship between the patterns of fetal growth and during the first year of life with lung function; thus, those children with restricted fetal weight growth had worse FEV_1_ independently of whether they caught-up or not compared to children with normal fetal and infant growth patterns. However, the authors appreciated that the growth pattern of accelerated fetal weight if it is followed by an accelerated growth pattern in infancy is accompanied by a significant increase in FVC and a decrease in the FEV_1_/FVC ratio without affecting the FEV_1_; therefore, disynaptic development with the accelerated weight gain is found. Unfortunately, this cohort does not report lung function measurements in the first months of life.
4. A metanalysis of lung function in 24 European cohorts compiling the data of 24,938 children checked that those with a greater monthly weight increase, in the first year of life, had a lower FEV_1_/FVC when their lung function was measured between the ages of 4 and 19 years, suggesting a disynaptic development of lung function in children with a greater weight gain during infancy (22).
5. Genetics. Some information regarding the prenatal determination of lung function has been contributed by genetic studies in recent years. Different single nucleotide polymorphisms (SNP) have been studied in different genes. Thus, in a cohort of 73 children recruited if one of their parents were atopic (28), a reduction in neonatal V_maxFRC_ was demonstrated in the children with the polymorphism *Gl*n^27^ in some allele in the beta-adrenergic receiver gene when compared with the polymorphism *Gl*u^27^*Glu* (95%CI of difference, −68 to −10; *p* = 0.011) and also in children with the SNP as *Ar*g^16^ in some allele compared with those that did not have *Ar*g^16^ (95% CI of difference −1.06 to −0.10; *p* = 0.02). Additionally, the 19 children of that cohort with the SNP *Ar*g^16^ and *Gl*n^27^ in some allele, had a clearly marked reduction in neonatal V_maxFRC_ compared to those that had no allele with such SNP (81 ± 36 mL/s vs. 145 ± 66; *p* < 0.001, respectively).

The influence of genetical variants was studied in the COPSAC cohort (29), which prior studies had demonstrated to be associated with adult lung function; to do so, 26 SNP were selected related with the FEV_1_/FVC ratio and eight SNP related with FEV_1_. The authors found no relationship with neonatal lung function but did find a relationship with the lung function measured at 7 years of age, thus suggesting that this group of genes is important in lung development, and opens a window of opportunity in these first years for interventions in the expression of these genes. They additionally did not find a relationship between different SNP of the Vascular Endothelial Growth Factor-A (VEGF-A) gene and neonatal lung function, although a relationship was found at school age (30), so it would seem that these genes also play a more important role in the development of post-natal lung function. However, some years before it had been described that the homozygotes CC of the rs3025028 SNP in the VEGF-A gene had significantly higher V'maxFRC and higher z-scores of FEF_50_ and FEF_25−75_ compared with other genotypes (31). The results cannot be considered contradictory since the SNP of the VEGF-A gene studied in both studies are different.

In the SWS cohort (32) the association of SNP was studied in four different genes and it was found that the rs1529672 SNP of the retinoic acid receptor b (RARB) gene and the rs12477314 of the histone deacetylase 4 (HDAC4) gene were associated with a greater V_maxFRC_.

The Perth cohort (33) studied the significant association of the A38G secretoglobin 1A1 (SCGB1A1) gene and, although they found no significant relationship with the V_maxFRC_, they did find it with bronchial hyperresponsiveness.

The finding of the interaction between intrauterine tobacco exposure and the expression of the Transferase genes (GSTT1, GSTP1) that are associated with a reduction in the V_maxFRC_ and to hyperresponsiveness in the exposed infants is particularly interesting (34).

## Does Asthma Itself Cause a Loss in Lung Function at an Early Stage of Life?

The data of cohorts that study the evolution of lung function from infancy to adulthood (4, 5, 35) show that patients with persistent asthma have a worse lung function both in adulthood as well as in infancy, but the differences with non-asthmatics do not change over time; this suggests that the loss occurs before the age at which the lung function was first evaluated in the cohorts (9 and 7 years, respectively).

The Tucson Children's Respiratory Study found a lower lung function in patients with persistent asthma at 6, 11, and 16 years(36), but not at 2 months of life; suggesting a structural change in the airways (airway remodeling) as a consequence of the asthma itself, before the age of 6 years. However, there does not seem to be any further drop with age with respect to the rest of the phenotypes nor with respect to those who never wheezed (36); therefore, the airway remodeling, if it indeed exists, must occur very early on in life (that is to say, before 6 years of age) and not progress later on in life.

However, the Perth cohort (10) showed that lung function was permanently low in persistent asthmatics in comparison with the non-asthmatic group, which suggests a congenitally-affected lung function. The data at 24 years of this cohort confirm this suggestion since the adult asthmatic patients showed a persistently worse lung function than the non-asthmatics (mean reduction: 16.2%; 95%CI 8.1 to 24.3%; *p* < 0.0001 considering V_maxFRC_ until 12 months and FEF_25−75_ from 6 years) (11). In this same cohort, the authors studied some risk factors for loss in lung function (12) and did not find that persistent asthma, in itself, reduced the lung function between 1 month and 18 years, although there seems to be a trend that is very near to statistical significance, since the percentage of change in the VmaxFRC-FEF_25−75_ related with the history of asthma was −6.0%; 95%IC −12.2 to +0.3; *p* = 0.061.

The authors of the COPSAC study (16) found that at 7 years the asthmatic patients, in comparison with the non-asthmatics, not only had had a lower lung function when they were neonates, but also suffered a progressive decrease in those 7 years in both FEF_50_ and in FEV_0.5_ (neonatal)/FEV_1_ (at 7 years). However, in the most recent publication on this cohort (17) when the children reached 13 years, the authors, with the aim of demonstrating whether there was a relationship between lung function loss and asthma development, did not find an interaction between asthma and the age that the measurement was performed, which suggests to them that the lung function in asthmatics is predetermined at a very early age, probably congenitally, and remains stable throughout infancy, even though the symptoms may cease. It is interesting that they also analyzed the change in lung function for each year of having asthma and found no significant variations; there do not seem to be any variations due to asthma remission either (Table 1). Using the authors own words (17): *The lung function deficit was present before the children developed asthma, did not progress with symptoms, and remained even if symptoms ceased*.

Therefore, with the exception of the TRCS cohort (36), the rest of the cohorts did not find the evolution of asthma to be a risk factor for losses in lung function and there seems to be a lower, but parallel, trajectory in lung function of asthmatics compared to healthy subjects, at least, until youth.

One interesting study assessed the pathological characteristics associated to airway remodeling in biopsies of bronchial mucosa, in children under the age of 2 years with recurrent wheezing compared with healthy children. No difference was found with regard to the thickness of the basal membrane, or in the number of inflammatory cells, and they concluded that said alterations are not present in symptomatic infants with reversible obstruction to the flow, even when they are atopic (37). Some years later, they reassessed the same biopsies when the patients had already reached 8 years and once again found no relationship between the thickness of the basal membrane nor the smooth bronchial muscle area with the diagnosis of asthma, lung function, nor atopic status, at that age (38). The results suggest that the pathological process initiated by asthma seems to be different from the eosinophilic inflammation and the airway remodeling characteristic of asthma in adults.

## Is the Development of Bronchial Hyperresponsiveness at an Early age a Risk Factor for Asthma?

Bronchial hyperresponsiveness (BHR) was assessed in the first months of life in some studies (17, 28, 39–43) with the aim of analyzing its relationship with the subsequent development of asthma (Table 2). The first article of this type was published in 1992 by Clark et al. (39), who evaluated BHR by means of increasing doses of inhaled histamine to determine the concentration that caused a 30% decrease in V_maxFRC_ (PC30) in 45 infants; 23 of whom had previously suffered lower respiratory tract infections (LRTI); and 22 had not. They did not find differences in the PC30 between the infants with LTRI and those without, which suggests that, at the age of 6 months, BHR does not discriminate between those with or without LTRI and, for those authors, the lower respiratory tract symptoms are not associated with the capacity of the bronchial response. That same cohort was studied at 10 years of age and the authors found no correlation between PC30 measured around 6 months of age with PC20 evaluated at 10 years of age; on the other hand, they found that a lower PC30 in infancy is a risk factor for suffering wheezing until 4 years, but that it is not so at 10 years of age (28) (Table 2). That is to say, the authors proposed that the early transient wheeze phenotype is related with BHR in the first months of life, but that early BHR is not associated with BHR nor wheezing at 10 years of age.

**Table 2:** Relationship between bronchial hyperresponsiveness (BHR) assessed in infants and asthma later in life.

**Author**	***N***	**Sample characteristics**	**Method**	**Age**	**Premorbid evaluation**	**Postmorbid evaluation**	**Main results**
Clarke JR et al. (39)	45	Cohort of newborn infants who had at least one atopic parent. Fifty percent had had previous lower respiratory tract infections	Increased doses of inhaled histamine by determining the provoking concentration which caused a 30% decrease in VmaxFRC (PC30).	6.5 months	50%	50%	There was no significant difference in PC30 between symptomatic infants (median 10.3 g/l; 95% CI 2–8 to 23–8 g/l) and control infants (median 16–5 g/l; 95% CI 2–4 to 27–9 g/l).
Wilson et al. (28)		Same cohort studied at 10 years					Neonatal BHR predict Lung function at 10 years (ß = 5.33; 95%CI 0.64 to 10.02; *p* = 0.03). Lower PC30 in infancy, is a risk factor for wheezing during the firsts 4 years of age (OR = 0.49; 95%CI 0.25 to 0.95; *p* = 0.03), but not at 10 years old.
Yao et al (40)	89	Infant suffering from Atopic dermatitis	The methacholine concentration that FEF_75_ by ≥30%.	10.7 months (range: 2.6–19.1)	100%		A lower PC30 (greater reactivity) was not a significant risk factor for increased number of wheezing episodes the year following the test.
Cox et al. (43)	203, 174, 147, 103, 176, and 137 at 1, 6, and 12 months and 6, 11, and 18 years of age, respectively	Perth Cohort recruited as newborns	BHR to histamine was assessed as a dose–response slope (DRS).	1, 6, and 12 months and 6, 11, and 18 years	100% at 1 month of age		Asthma at 18 years was associated with increased airway responsiveness at 6, 12, and 18 years (At 6 years: slope 0.24; 95%CI 0.06 to 0.42; *p* = 0.01. At 12 years: slope 0.25; 95%CI 0.08 to 0.49; *p* = 0.006. At 18 years: slope 0.56; 95%CI 0.29 to 0.83; *p* < 0.001). However, asthma was not associated with BHR during infancy (At 1 month: slope −0.07; 95%CI −0.19 to −0.04; *p* = 0.21. At 6 months: slope −0.08; 95%CI −0.21 to −0.05; *p* = 0.21. At 12 months: slope −0.06; 95%CI −0.18 to −0.07; *p* = 0.36).
Bisgaard et al. (16)	411	COPSAC Cohort recruited as newborns whose mothers had been diagnosed from asthma	Provocative dose of Methacholine that cause a 15% fall of transcutaneous saturation of oxygen (PD15-PtcO2).	1 month	100%		Bronchial responsiveness to methacholine in the neonates was associated with the development of asthma at 7 years old (OR per interquartile range: 1.59; 95%CI: 1.11–2.28; *p* < 0.01).
Hallas et al. (17)		The same cohort evaluated at 13 years old					There was an increased reactivity to methacholine in neonates who developed asthma in their first 13 years of life compared with never diagnosed from asthma (Difference in PD_z_ *z*-score = −0.40; 95%CI −0.58 to −0.22); *p* < 0.001.

The BHR was assessed in a cohort of children diagnosed with atopic dermatitis, by means of the concentration of methacholine that could decrease the forced expiratory flow at 75% of the expired volume (FEF_75_) by ≥30%, at an age range of between 2.6 and 16.9 months (40). The methacholine test was carried out before any episode of wheezing in all the infants. The authors' conclusion was that a higher BHR is not a risk factor for a greater number of wheezing episodes in the year following the test (*p* > 0.12).

The Perth cohort also evaluated the relationship between BHR, by means of the dose-response slope, and the subsequent development of asthma (41–43). They performed the BHR test at 1 month of life, at 6 and 12 months and also at 6, 11, and 18 years of age. The authors concluded that the asthma at 18 years was associated with BHR measured at 6 years (*p* = 0.01), 12 years (*p* = 0.006), and 18 years (*p* < 0.001) but not with that determined during infancy; i.e., at 1 month (*p* = 0.21), 6 months (*p* = 0.21), and 12 months (0.36) (43). The authors proposed that the BHR during infancy may reflect the initial geometry of the airways in the first years of life, but that from school age BHR could be a consequence of the effect of the immunological and environmental factors on the airways and, therefore, the response capacity of the airways measured later on in infancy becomes a better predictor of future asthma (43).

All these studies concur in considering that, although BHR may be present very early on in infancy, it bears no association on future asthma. In fact, they concur with the study by Montgomery and Tepper (44) in healthy infants, in which the authors described the decrease in sensitivity to methacholine as age increases.

The Copenhagen cohort, on the other hand, shows that BHR evaluated at 1 month of life is a risk factor for asthma at 7 years of age (Table 2) (16). These results were corroborated shortly after, when the participants of the cohort had reached 13 years (17), in such a way that the asthmatics had presented greater BHR at 1 month of life compared to the non-asthmatics. For the authors, this suggests that there are some inherent and stable characteristics, not caused by inflammation during symptomatic periods, that predispose to developing asthma, hyperresponsiveness, and intermittent bronchial obstruction (17).

## Conclusions

In children with persistent asthma the lung function seems to be reduced at a very early age and remains low throughout their whole adult life. This loss so early on is probably a congenital characteristic, although factors such as prematurity, fetal, and immediate postnatal growth as well as exposure to tobacco smoke may also be important deleterious factors.

There are contradictory results as to whether asthmatic children lose lung function as a consequence of airway remodeling due to the disease itself; but the majority of the cohort studies find a lower but parallel path of lung function in asthmatics compared to non-asthmatics.

Bronchial hyperresponsiveness measured in the first months of life seems to be very frequent even in healthy infants, but is probably not a risk factor for asthma years later, and is only a risk factor for wheezing when it appears at pre-school age.

Great uncertainty still abounds in this field; therefore, further research is needed to better understand the physiopathology of asthma, its early risk factors and to design new therapeutic goals and early interventions to change the natural history of the disease.

## Author Contributions

The author confirms being the sole contributor of this work and has approved it for publication.

### Conflict of Interest Statement

The author declares that the research was conducted in the absence of any commercial or financial relationships that could be construed as a potential conflict of interest.
